# Wild Edible Fruit of *Prunus nepalensis* Ser. (Steud), a Potential Source of Antioxidants, Ameliorates Iron Overload-Induced Hepatotoxicity and Liver Fibrosis in Mice

**DOI:** 10.1371/journal.pone.0144280

**Published:** 2015-12-03

**Authors:** Dipankar Chaudhuri, Nikhil Baban Ghate, Sourav Panja, Abhishek Das, Nripendranath Mandal

**Affiliations:** Division of Molecular Medicine, Bose Institute, P 1/12, C. I. T. Road, Scheme–VIIM, Kolkata, 700054, West Bengal, India; Northeastern University, UNITED STATES

## Abstract

The antioxidant and restoration potentials of hepatic injury by *Prunus nepalensis* Ser. (Steud), a wild fruit plant from the Northeastern region of India, were investigated. The fruit extract (PNME) exhibited excellent antioxidant and reducing properties and also scavenged the 2,2-diphenyl-1-picrylhydrazyl (DPPH) radical (IC_50_ = 30.92 ± 0.40 μg/ml). PNME demonstrated promising scavenging potency, as assessed by the scavenging of different reactive oxygen and nitrogen species. Moreover, the extract revealed an exceptional iron chelation capacity with an IC_50_ of 25.64 ± 0.60 μg/ml. The extract induced significant improvement of hepatic injury and liver fibrosis against iron overload induced hepatotoxicity in mice in a dose-dependent manner, and this effect was supported by different histopathological studies. The phytochemical constitutions and their identification by HPLC confirmed the presence of purpurin, tannic acid, methyl gallate, reserpine, gallic acid, ascorbic acid, catechin and rutin. The identified compounds were investigated for their individual radical scavenging and iron chelation activity; some compounds exhibited excellent radical scavenging and iron chelation properties, but most were toxic towards normal cells (WI-38). On the other hand, crude PNME was found to be completely nontoxic to normal cells, suggesting its feasibility as a safe oral drug. The above study suggests that different phytochemicals in PNME contributed to its free radical scavenging and iron chelation activity; however, further studies are required to determine the pathway in which PNME acts to treat iron-overload diseases.

## Introduction

An imbalance between the efficiency of body's antioxidant defense and the free radical generation system, involving reactive oxygen species (ROS) and reactive nitrogen species (RNS), eventually gives rise to oxidative stress. These reactive species are mainly responsible for the primary initiation of oxidative damage to several important biological macro-molecules, like membrane lipids, proteins and nucleic acids [1]. Increasing evidence indicates that redox-active metals such as iron also play a major role in the overproduction of ROS by undergoing redox cycling [2]. Although the transition metal iron is essential for important biological activities and biochemical reactions, an excess of iron is toxic, presumably via formation of highly reactive hydroxyl radicals through the Fenton reaction causing lipid peroxidation, depletion of low-molecular weight antioxidants, DNA alterations, hemochromatosis, L-thalassemia, ischemic heart disease and cancer [3–4]. Therefore, substances that are able to trap ‘free iron’, making it unavailable for Haber-Weiss reactions, act as antioxidants [3]. In addition to endogenous antioxidant systems, frequent consumption of foods rich in natural antioxidants also results in increased resistance to oxidative stress by altering the redox environment and is associated with a lower risk of many oxidative stress-related diseases.

In recent times, edible fruits have attracted substantial interest because they contain several antioxidants and bioactive phytocompounds that may act as possible remedial agents. An important plant of interest is *Prunus nepalensis* Ser. (Steud) (family, Rosaceae), grown in different parts of Northeast India. Locally known as *Sohiong* in East Khasi hills of Meghalaya, the edible fruits of *P*. *nepalensis* are often used for making fruit juice, jam, squash and assorted wines. Moreover, the fruits of *P*. *nepalensis* are used as an astringent, the leaves are a diuretic agent and are used for edema [5]. Several bioactive compounds, such as quercetin, quinic acid, rutin, scopoletin, naringenin, palmitoleic acid and many others, have been isolated from different species of *Prunus* available in China [6]. However, only a few studies have focused on the preliminary quantification of phenolic compounds and antioxidant capacity of *P*. *nepalensis* [7]. A review of the literature showed that the detailed phytochemical composition, antioxidant activity and recovery effects from hepatic toxicity by aqua-methanolic extract of the fruits on iron-induced liver damage remain unexplored.

Thus, this study was performed to determine the phytochemical profile and the *in vitro* and *in vivo* antioxidant properties of 70% methanolic extract of *P*. *nepalensis* fruit (PNME) and *in vivo* amelioration of liver toxicity due to iron overload in Swiss albino mice.

## Materials and Methods

### Chemicals and reagents

ABTS [2,2′-azinobis-(3-ethylbenzothiazoline-6-sulfonic acid)], 6-hydroxy-2,5,7,8-tetramethylchroman-2-carboxylic acid (Trolox) was purchased from Roche diagnostics (Mannheim, Germany) and Fluka (Buchs, Switzerland), respectively. Nitro blue tetrazolium (NBT), 2-deoxy-2-ribose, sodium nitroprusside (SNP), phenazine methosulfate (PMS), 1,10-phenanthroline, 5,5′-dithiobis-2-nitrobenzoic acid (DTNB), lipoic acid, ferrozine and bathophenanthrolinesulfonate disodium salt were purchased from Sisco Research Laboratories Pvt. Ltd, Mumbai, India. Folin-ciocalteu reagent, 2,4-dinitrophenylhydrazine (DNPH), butylated hydroxyltoluene (BHT), 1-chloro-2,4-dinitrobenzene (CDNB), chloramine-T, Dimethyl-4-aminobenzaldehyde and hydroxylamine hydrochloride were taken from Merck, Mumbai, India. Spectrochem Pvt. Ltd, (India) supplied Diethylenetriaminepentaacetic acid (DTPA). Iron-dextran and guanidine hydrochloride was obtained from Sigma-Aldrich, USA. Desirox (Deferasirox) was purchased from Cipla Ltd., India. All the other used reagents are molecular biology grade and obtained from reputed suppliers.

### Ethics

The fresh fruits of *P*. *nepalensis* were collected in October 2013 from local neighboring villages in Shillong (25.6314° N, 91.8840° E), Meghalaya, India. Collection zones are not under a Government-protected area, National Park or Reserve Forest and the collection was performed only after receiving oral permission from the village headmen. The International Union for Conservation of Nature (IUCN), World Conservation Union guidelines (1994) was used to classify the conservation status and was found that this plant has not yet been assessed for the IUCN Red List.

*In vivo* experiments were performed abiding by the guidelines of the Committee for the Purpose of Control and Supervision of Experiments on Animals (CPCSEA), Ministry of Environment and Forest, Govt. of India with due approval from the Institutional Animal Ethics Committee, Bose Institute (Registration. No. 95/1999/CPCSEA). All surgeries were done using ethyl ether as anesthetic (inside an appropriate fume hood), taking utmost care to reduce suffering.

### Test Animals

Swiss albino mice (Male, weighing 20 ± 2 g) were acquired from Chittaranjan National Cancer Institute (CNCI), Kolkata, India. The animals were kept under a continuous 12 h light / dark cycle (temperature-22 ± 2°C). The animals were fed with laboratory diet and water *ad libitum*. Experimental animals were taken care every 6 h during the treatment period and it was observed that there was no unwanted animal death.

### Fruit Extract Preparation

*P*. *nepalensis* was authenticated by the Botanical Survey of India, Eastern regional center, Shillong, Meghalaya, India (Acc. No. 52320, 45246). The collected fruits were crushed to separate the seeds from the pulp. The finely ground pulp (100 g) was mixed with 1000 ml of methanol:water (7:3) at 37°C overnight using a shaking incubator. The mixture was then centrifuged at 2850 *g*, and the supernatant was collected. The pellet was again suspended in 1000 ml of the same solvent and the procedure was repeated. The obtained supernatants were concentrated in a rotary evaporator under reduced pressure followed by lyophilization. The lyophilized powder was labeled as PNME and stored at -20°C. Water was used as the vehicle/solvent of choice for further experiments, as PNME dissolves easily in water.

### In vitro Antioxidant and free radical scavenging activity

#### Antioxidant activity

ABTS^•+^ radical cation decolorization assay was used to evaluate the antioxidant capacity of PNME (0.05–10 mg/ml) with respect to the standard, trolox [8]. Standard methods were used to evaluate the Fe^3+^-reducing capacity of the extract [8]. The DPPH (2,2-diphenyl-1-picrylhydrazyl) scavenging potential of the extract was investigated according to Ghate *et al*, 2013 [9]. The scavenging percentage of PNME was evaluated from the values of the control and the test samples.

#### ROS scavenging assays

The ROS scavenging activity of the extract was revealed by several *in vitro* radical scavenging assays, such as superoxide, hydroxyl, hypochlorous radical and singlet oxygen assays, using standard procedures [8].

#### RNS scavenging assays

The RNS scavenging potentials of PNME was evaluated by performing nitric oxide and peroxynitrite radical scavenging assays [8].

#### Metal chelating activity

The Fe^2+^ chelating ability was determined as described earlier [8]. Protection efficacy of PNME in Fe^2+^-mediated supercoiled plasmid DNA (pUC18) breakdown was determined following previously described methods [9], with slight modifications. The densitometric analysis of the DNA bands was performed using ImageJ 1.47v tool by the software company NIH, USA. The DNA supercoil protection capacity of the fruit extract was expressed as the [P]_50_ value, the amount of sample required for 50% protection. The degree of inhibition of Fe^2+^-mediated lipid peroxidation was assessed by estimating the TBARS by the method of Ghate et al. 2013 [9] using freshly prepared brain homogenates of Swiss albino mouse brains. The results are expressed as a percentage of inhibition.

### In vivo hepato-ameliorating activity

#### Experimental design and tissue preparation

Six groups were randomly created comprising six mice each. Among them, one group was labeled the blank (B) and was administered saline. The remaining groups were injected intraperitoneally (ip) with five doses of iron-dextran of 100 mg/kg b.w. (one dose every alternative days). After the first injection oral treatment was started the next day with only saline to the iron-dextran group (C), and the remaining groups were treated with 50 mg/kg b.w. PNME (S50), 100 mg/kg b.w. PNME (S100), 200 mg/kg b.w. PNME (S200) or 20 mg/kg b.w. desirox (D) for the 21 successive days. All experimental animals were sacrificed on 22^nd^ day under mild anesthesia (ethyl ether) and cardiac puncture was performed to collect blood and serum was separated and stored at -80°C. After collecting the blood, the liver was quickly excised, cleaned thoroughly with cold phosphate buffer saline (PBS) to remove the remaining blood and cut into three sections. The major liver portion was dissected and homogenized using 10 volumes of 0.1 M phosphate buffer (pH 7.4) supplemented with 0.15 M NaCl and 5 mM EDTA and centrifuged for 30 min at 8000 *g* in the cold. The clear homogenate (supernatant) was collected and the protein concentration was quantified by Folin-Lowry method [10], where BSA was used as a standard; the remaining supernatant was then stored at -80°C. Second liver fragment was treated with a mixture of nitric acid and sulfuric acid (1:1) to analyze the iron content. The remaining portion was processed for histopathological examinations.

#### Serum Markers

Aspartate amino transferase (ASAT), alanine amino transferase (ALAT), and billirubin levels in the serum were evaluated using commercially available kits from Merck (India). Moreover, alkaline phosphatase (ALP) levels in the serum were measured by a kit procured from Sentinel Diagnostics, Italy.

#### Antioxidant enzymes

The suppression of the blue-colored formazan formation was assessed at 560 nm to evaluate superoxide dismutase (SOD) levels [11]. Catalase (CAT) activity was assessed by tracking the breakdown of H_2_O_2_ over time at 240 nm [12]. Glutathione-S-transferase (GST) levels were estimated based on the formation of GSH-CDNB conjugate and increase in absorbance at 340 nm [13]. The level of reduced glutathione (GSH) was determined spectrophotometrically at 412 nm [14].

#### Evaluation of liver injury

The products of lipid peroxidation in liver were quantified as thiobarbituric acid reactive substances (TBARS) [15]. Protein carbonyl content was estimated spectrophotometrically to determine the levels of protein oxidation [16]. The measurement of hydroxyproline content in the liver allows for the quantification of collagen content, which is an important marker of liver fibrosis. The respective homogenates were hydrolyzed in 6 M HCl and hydroxyproline was measured by Ehrlich’s solution [17]. The absorbance was taken at 558 nm and the results were calculated from standard curve of 4-hydroxy-L-proline (*R* ^*2*^ = 0.9907). The collagen content in each sample was determined by multiplying the factor of 7.69 with the quantity of overall hydroxyproline content [18].

#### Serum ferritin and liver iron

The manufacturer’s instructions were followed to quantify serum ferritin levels using an enzyme-linked immunosorbent assay (ELISA) kit (Monobind Inc., USA). Iron content in liver was quantified using a previously reported method [19]. Briefly, samples were mixed with bathophenanthroline sulfonate and incubated at 37°C for 30 min and absorbance was recorded using a spectrophotometer at 535 nm.

#### Histopathological investigation

PBS washed excised liver samples were fixed for two days in 10% buffered neutral formalin. Sections (5-μm thick) were cut using the paraffin-embedding technique and stained with hematoxylin and eosin (morphological examination), Masson’s trichrome stain (liver fibrosis) and Perls’ Prussian blue dye (iron content). The stained sections were checked microscopically for changes in histopathological condition.

#### In vitro ferritin iron release

Iron reduction and release were determined using ferrozine, a spectrophotometric reagent for iron, as previously described [20]. Briefly, the reaction was initiated by adding different concentrations (100–500 μg/ml) of PNME in 50 mM phosphate buffer (pH 7.0) containing 200 μg of ferritin and 500 μM ferrozine and the absorbance change was measured for 20 min at 560 nm. The reaction mixture excluding PNME was used as a reference.

### Identification of active phytochemicals and standardization of the extract

The evaluation of existing phytochemicals, such as phenols, carbohydrates, alkaloids, flavonoids, glycosides, ascorbic acid, tannins, saponins, terpenoids, triterpenoids and anthraquinones, in PNME was performed using standard methods [21–22]. Among the present phytochemicals phenols, carbohydrates, flavonoids, alkaloids, tannins, ascorbic acid were quantitatively analyzed following previously described methods [9,23]. Extract standardization was performed by HPLC analysis [24]. The samples were eluted using acetonitrile and 0.5 mM ammonium acetate in water as a mobile phase for gradient elution (flow rate of 1 ml/min) through the column (ZIC-HILIC) for 80 min at 25°C and the peaks were detected at 254 nm.

### Cytotoxicity assay

The human normal lung fibroblast cell line (WI-38) was procured from the National Centre for Cell Science (NCCS), Pune, India. Cells were cultured in Dulbecco's Modified Eagle Medium (DMEM) provided with 10% (v/v) fetal bovine serum (FBS), 50 μg/ml gentamicin sulfate, 2.5 μg/ml amphotericin B, 100 U/ml penicillin G, 100 μg/ml streptomycin and maintained in a biological CO_2_ incubator. Cell viability was calculated using the WST-1 Cell Proliferation Reagent (Roche Diagnostics) as described previously [25]. Briefly, the cells (1 × 10^4^ cells/well) were incubated in presence of PNME, purpurin, tannic acid, reserpine, methyl gallate, catechin, ascorbic acid, gallic acid or rutin for 48 hours with increasing doses from 0–120 μg/ml in 96-well culture plates. A total of 10 μl of the reagent was then added to each well and the samples were kept at 37°C for 2 hours. Absorbance (460 nm) was measured using a microplate ELISA reader MULTISKAN EX (Thermo Electron Corporation, USA) to calculate cell proliferation and viability.

### Statistical analyses

All data are reported as the mean ± S.D. (n = 6). Statistical analysis was performed using Origin professional 6.0 and KyPlot version 2.0 beta 15 (32 bit). Comparisons between the groups were evaluated according to paired *t*-test, and a *p* value of <0.05 was considered significant.

## Results and Discussion

Epidemiological studies have established that consumption of plant-based foods containing abundant sources of antioxidants is beneficial for health because these antioxidants obstruct many degenerative processes and effectively lower the incidence of oxidative stress-related diseases [26]. Antioxidants may also act as iron chelating agents because many of them consist of a range of bidentate, tridentate and hexadentate ligands in which two, three, or six atoms, respectively, are able to coordinate with iron, forming octahedral complexes [27] followed by their excretion from the body. With the intention to investigate the fundamental principle of traditional usage of the fruits as medicine, biologically active phytocomponents present in the plant need to be well extracted with different solvents. Polar solvents are frequently employed for the recovery of polyphenols from a plant matrix. Generally, extraction of bioactive compounds from various biological resources (whole plants, fruits, vegetables, etc.) is performed using aqueous mixtures (hot or cold) comprising methanol and ethanol [28,29]. Keeping this in mind, extraction of the phytochemicals from the *P*. *nepalensis* fruit pulp was performed using 70% methanol at room temperature.

### In vitro antioxidant potential of PNME

#### Total antioxidant capacity

**Fig 1:**
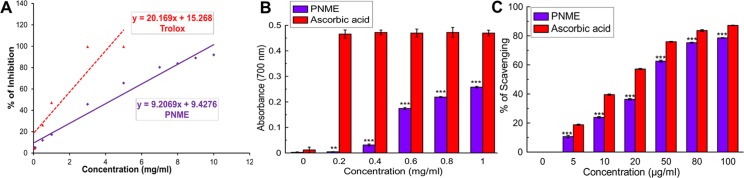
Antioxidant activity of PNME. **A.** Total antioxidant assay, **B.** Reducing power assay, **C**. DPPH scavenging assay. The results are the mean ± S.D. (n = 6). **p < 0.01 and ***p < 0.001 vs. control.

The antioxidant activity of PNME was measured using two complementary methods: TEAC through ABTS^•+^ radical cation scavenging and reducing power capacity, along with the scavenging assay of DPPH. A strong correlation between antioxidant activity and reducing power was revealed previously [30]. On the other hand, DPPH in its radical form has its maximum absorbance at 517 nm, but upon reduction with an antioxidant, its net absorbance decreases due to the formation of its non‐radical form, DPPH–H [31]. The assays project an overall indication of the antioxidant potential of the fruit extract as shown in Fig 1A, 1B and 1C along with Table 1. Total antioxidant activity, a function of the trolox (standard)-equivalent antioxidant capacity (TEAC) of PNME was observed to be 0.46 ± 0.001. PNME can be considered a potent antioxidant agent for its exceptional DPPH radical scavenging activity (Fig 1C), in spite of its moderate reducing capacity (Fig 1B).

**Table 1:** IC_50_ values of the PNME and standard compounds for different antioxidant, free radical and metal chelation assays.

Name of Assay	PNME	Standards	Values of Standard compounds
DPPH	30.92 ± 0.40	Ascorbic acid	5.29 ± 0.28
Superoxide anion (O_2_•^−^) scavenging	225.73 ± 3.09	Quercetin	42.06 ± 1.35
Hydroxyl radical (OH•) scavenging	1162.99 ± 23.05	Mannitol	571.45 ± 20.12
Hypochlorous acid (HOCl) scavenging	309.68 ± 18.15	Ascorbic acid	235.96 ± 5.75
Singlet oxygen (^1^O_2_) scavenging	743.44 ± 13.87	Lipoic acid	46.15 ± 1.16
Nitric oxide radical (NO) scavenging	321.25 ± 4.15	Curcumin	90.82 ± 4.75
Peroxynitrite (ONOO-) scavenging	2.25 ± 0.42	Gallic acid	0.876 ± 0.57
Iron chelating activity	25.64 ± 0.60	EDTA	1.27 ± 0.05
Lipid peroxidation	263.75 ± 12.11	Trolox	6.76 ± 0.17

All the values are expressed in μg/ml excluding in Peroxynitrite scavenging assay where values express in mg/ml. Data expressed as mean ± S.D (n = 6). EDTA represents Ethylenediamine tetraacetic acid.

#### ROS scavenging

**Fig 2:**
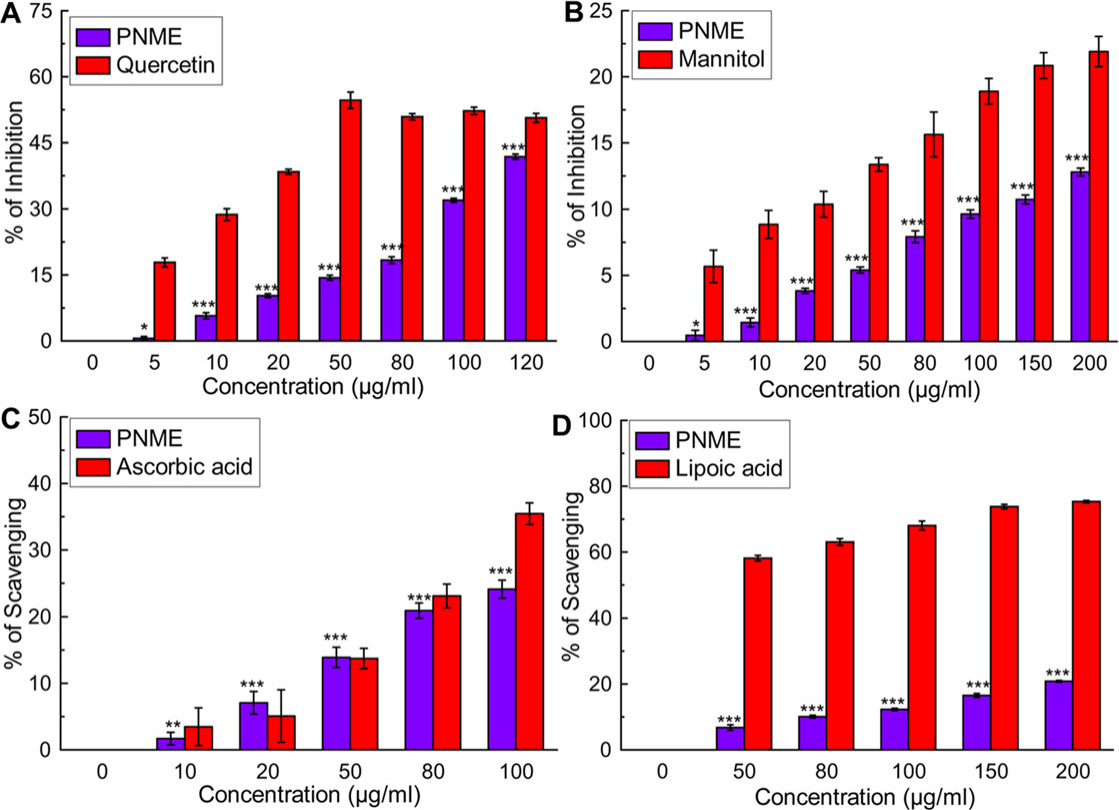
ROS scavenging activity of PNME. **A.** Superoxide radical inhibition, **B.** Hydroxyl radical inhibition, **C**. Hypochlorous radical scavenging, **D.** Singlet oxygen radical scavenging. The results are the mean ± S.D. of six parallel measurements. *p < 0.05, **p < 0.01 and ***p < 0.001 vs. control.

Various ROS, such as the superoxide anion, hydroxyl radical and hypochlorous acid, are generated under numerous conditions *in vivo*. It is well known that superoxide anion radical primarily initiates the formation of other ROS [32]. Superoxide radicals react with cellular H_2_O_2_ and generate the detrimental hydroxyl radical, which damages lipids, DNA, and proteins [8]. In the presence of H_2_O_2_, another harmful ROS, HOCl is produced *in vivo* by the oxidation of Cl‾ ions catalyzed by neutrophil-derived myeloperoxidase at sites of inflammation [33]. HOCl inactivates catalase, an antioxidant enzyme causes sulfhydryl oxidation in the plasma membrane proteins to induce target cell lysis [34]. On the other side, a singlet oxygen radical is generally produced *in vivo* by photo-excitation upon exposure to UV radiation or by chemi-excitation. This radical also inactivates antioxidant enzymes and induces hyper-oxidation and oxygen cytotoxicity [35]. PNME showed excellent dose-dependent scavenging activities against superoxide radical as well as hypochlorous acid (Fig 2A and 2C respectively). At the same time, PNME moderately scavenged hydroxyl radical in conjunction with singlet oxygen radicals (Fig 2B and 2D respectively). The IC_50_ values of PNME on different radical scavenging assays are shown in Table 1 along with their corresponding standard compounds.

#### RNS scavenging

**Fig 3:**
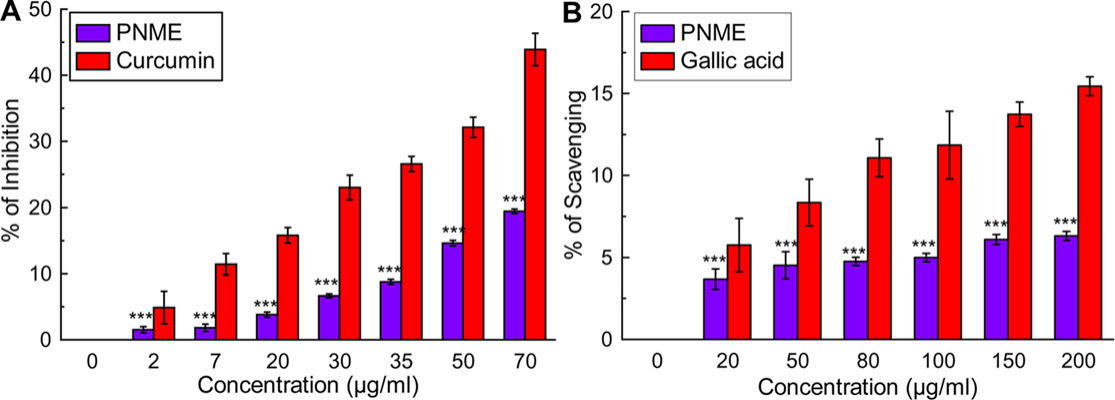
RNS scavenging activity of PNME. **A.** Nitric oxide inhibition, **B.** Peroxynitrite radical scavenging. The results are the mean ± S.D. of six parallel measurements. ***p < 0.001 vs. control.

In normal cellular conditions, nitric oxide is essential in various inflammatory processes but also contributes to multiple sclerosis, reperfusion injury, ulcerative colitis and arthritis, etc., when produced in excess amounts [36]. The impact of NO^•^ toxicity is critically amplified when superoxide radical reacts with it to form peroxynitrite anion [ONOO‾], which is highly reactive [37]. PNME moderately scavenges both RNS forms (Fig 3). The IC_50_ values of PNME for both the radicals are displayed in Table 1 with their corresponding standard compounds.

#### Metal chelation activity

**Fig 4:**
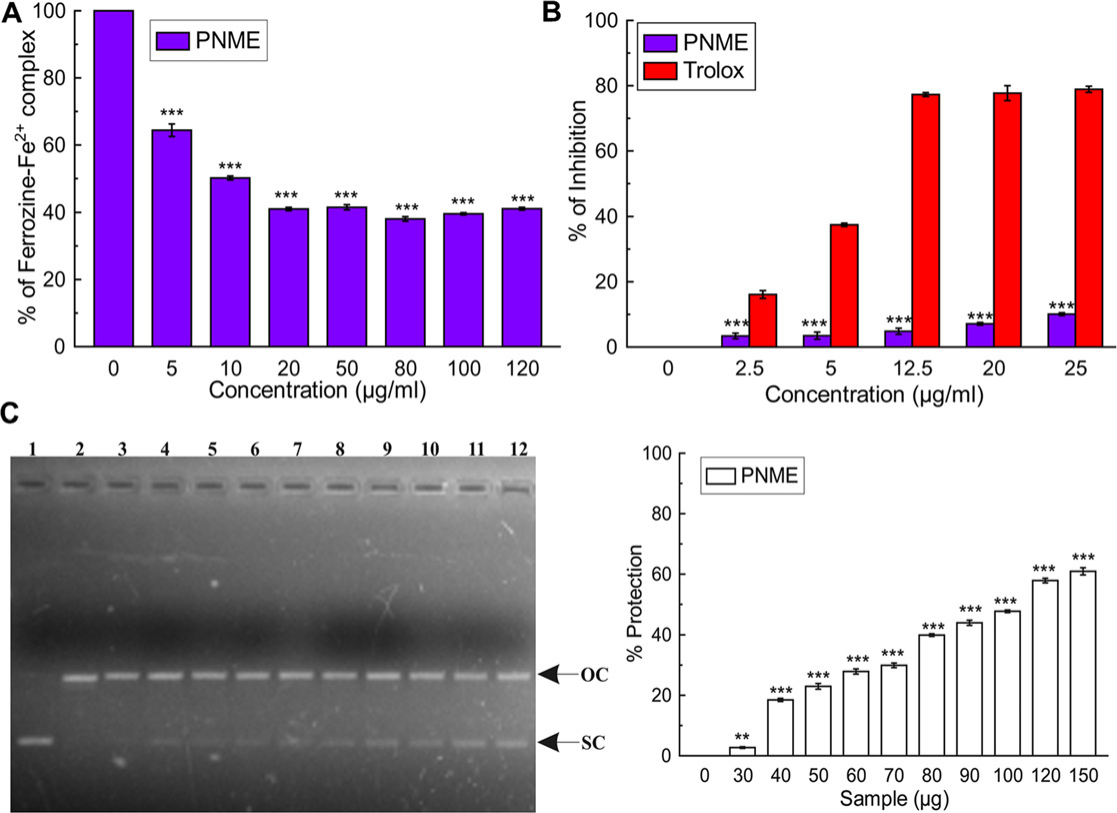
Metal chelation potential of PNME. **A.** Iron chelation assay, **B.** Inhibition of lipid peroxidation, **C.** DNA protection assay. Agarose gel showing bands of supercoiled (SC) and open circular (OC) forms of pUC-18 DNA, and the graph denotes the % protection of supercoiled DNA by PNME. The results are the mean ± S.D. (n = 6). **p < 0.01 and ***p < 0.001 vs. control.

Many antioxidants not only convert free radicals to more stable products but also slow the rate of oxidation by chelation of pro-oxidant metals (such as iron, copper, etc.) that promote oxidation by acting as catalysts of free radical reactions. Metal chelation by certain compounds decreases their pro-oxidant effect by reducing their redox potentials and also through steric hindrance by forming a metal hydroperoxide complex [38]. The iron chelation ability of the extract was revealed using three *in vitro* assays depending on the direct chelation of iron by the extract. Fig 4A suggests impressive results from PNME, as did the obtained IC_50_ value, indicating the extract’s effective iron chelating activity. In contrast, the most detrimental OH^•^, generated by the Fenton reaction (H_2_O_2_ + Fe^2+^ = Fe^3+^ + OH‾ + OH^•^) destroyed the lipid membrane by lipid peroxidation [39]. The extract moderately inhibited lipid peroxidation in a dose-dependent manner (Fig 4B) compared with standard compounds, although PNME showed impressive IC_50_ values (Table 1). Overall, PNME was found to be well endowed with exceptional iron chelation ability. The studies on the protective effect of PNME against OH^•^-mediated plasmid DNA (pUC18) slicing was also displayed a significant dose dependence (Fig 4C). An impressive [P]_50_ value of 129.56 ± 1.73 μg/ml was obtained, further supporting this notion.

### In vivo antioxidant and hepato-ameliorating activity

The liver, which is involved in numerous biochemical pathways related to nutrition and detoxification [40], is often subjected to injuries induced by various hepatotoxins. Iron, a vital constituent of countless proteins [41] becomes a well-known hepatotoxin when in excess. In iron-overloaded liver, the metal initiates and propagates several ROS, leading to the oxidative damage of many vital biomolecules, resulting in the cellular lipid peroxidation, mitochondrial damages, DNA fragmentation and, finally, cell death [42]. An effective remedial strategy should act in a dual manner by decreasing the oxidation rate: one manner sequestering and chelating the stored iron in cells [43] and other as a radical scavenger (i.e., antioxidant activity). Because PNME depicted excellent antioxidant and free radical scavenging activities along with significant *in vitro* iron chelation potency, the *in vivo* ameliorating potency of PNME on accumulation of iron and oxidative damage by iron overload in the mouse liver was studied. The hemochromatosis condition was created by the intraperitoneal injection of iron-dextran. This process will not hamper the intestinal iron absorption by fruit extract, which ultimately leads to iron overload in the liver as well as in serum [44].

#### Serum marker enzymes

Administration of excess iron dextran caused significant liver damage leading to the release of intracellular enzymes into the blood [45], as evidenced by the elevated level of serum parameters (Table 2). However, PNME treatment at a dose of 200 mg/kg induced a marked restoration of liver enzymes and bilirubin. Among them, the ALAT and ASAT values obtained from the PNME group were substantially superior to those from the standard drug desirox.

**Table 2:** The changes in ALAT, ASAT, ALP and Bilirubin levels after oral therapy with PNME.

Treatment	ALAT		ASAT		ALP		Bilirubin	
	Unit/L	% change	Unit/L	% change	Unit/L	% change	mg/dl	% change
**B**	15.25±1.58		67.61± 5.78		33.08±3.71		1.51±0.09	
**C**	45.23±2.45^X3^	196.63	167.62±4.64 ^X3^	147.92	132.51±6.42 ^X3^	300.58	3.28±0.20 ^X3^	116.29
**S50**	39.52±1.21 ^X3Y2^	159.16	129.01±9.30 ^X3^	90.82	123.72±3.59 ^X3Y1^	274.01	3.06±0.19 ^X3^	101.36
**S100**	28.82±2.26 ^X3Y3^	88.99	99.81±3.10 ^X3 Y3^	47.63	99.43±6.27 ^X3Y3^	200.58	2.85±0.30 ^X3Y2^	87.78
**S200**	19.21±2.18 ^X1Y3^	25.95	75.18±4.81 ^Y3^	11.19	66.35±6.47 ^X3Y3^	100.58	2.61±0.22 ^X3Y2^	71.95
**D**	23.55±1.09 ^X3Y3^	52.80	79.35±4.37 ^X1 Y3^	17.36	61.01± 2.44 ^X3Y3^	84.44	1.68 0.15^Y3^	10.97

Values are mean ± SD (n = 6). X1: p<0.05, X2: p<0.01 and X3: p<0.001 significant difference from B group. Y1: p<0.05, Y2: p<0.01 and Y3: p<0.001 significant difference from C group

#### Effect on antioxidant enzymes

**Fig 5:**
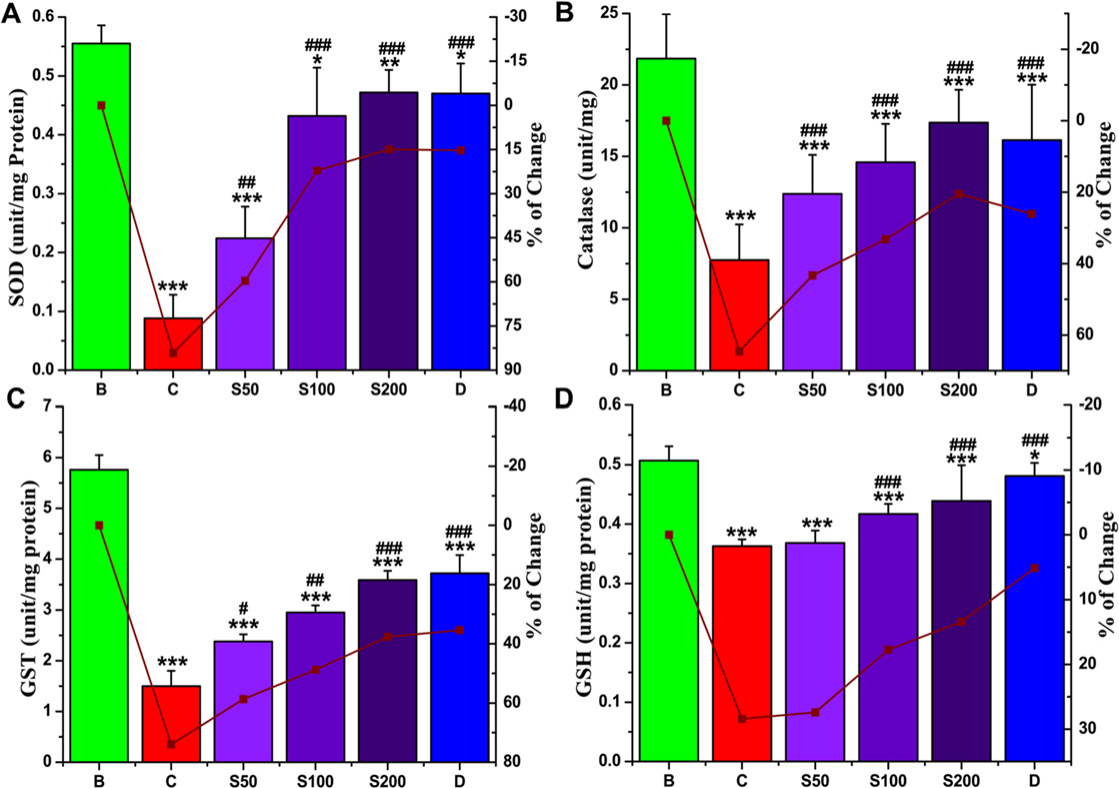
Effect of PNME on liver antioxidant enzymes level. **A.** SOD, **B.** Catalase, **C**. GST, **D.** GSH. Mouse groups (blank, B; control, C; 50 mg/kg b.w. PNME, S50; 100 mg/kg b.w. PNME, S100; 200 mg/kg b.w. PNME, S200; desirox group, D) were treated as described in the ‘Experimental design and tissue preparation’ section. The values are expressed as the mean ± S.D. (n = 6). *p < 0.05, **p≤ 0.01, ***p≤ 0.001 compared with blank, and ^#^p < 0.05, ^##^p≤ 0.01, ^###^p≤ 0.001 compared with control.

Living systems, especially animals, are armed with free radical scavenging enzymes, such as SOD, CAT, GST and the small compound GSH, which are the primary defense against oxidative damage. Levels of these antioxidant enzymes indirectly indicate the pro-oxidant-antioxidant condition in tissues [46]. A significant reduction in antioxidant enzymes levels was detected in iron-intoxicated mice compared with normal mice. PNME treatment significantly increased antioxidants levels, establishing its ameliorating potential against iron-overloaded oxidative stress. It was observed that, administration of the highest extract dose resulted in levels approaching those obtained from the standard desirox, in case of SOD (Fig 5A) and GST (Fig 5C), but not GSH (Fig 5D). Moreover, with regard to CAT (Fig 5B), the extract exhibited activity superior to the standard.

#### Biochemical parameters of liver damage

**Fig 6:**
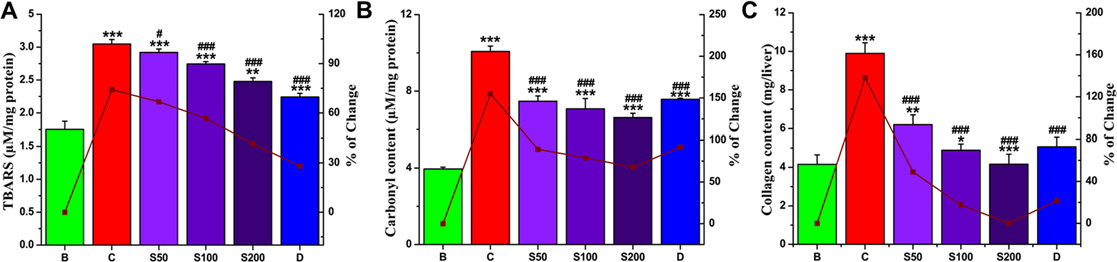
Effect of PNME on the liver damage parameters. **A.** Lipid peroxidation level, **B.** Protein carbonyl content, **C**. Hydroxyproline content. Mouse groups (blank, B; control, C; 50 mg/kg b.w. PNME, S50; 100 mg/kg b.w. PNME, S100; 200 mg/kg b.w. PNME, S200; desirox group, D) were treated as described in ‘Experimental design and tissue preparation’ section. The values are expressed as the mean ± S.D. (n = 6). *p < 0.05, **p≤ 0.01, ***p≤ 0.001 compared with blank and ^#^p < 0.05, ^###^p≤ 0.001 compared with control.

Lipid peroxidation (LPO) has been proposed as a major factor in iron toxicity, including iron-induced hepatotoxicity. Ferrous salts undergo the Fenton reaction to form the highly reactive hydroxyl radical, which attacks all biological molecules, including cell membrane lipids, to initiate LPO [47]. Iron-overloaded liver pathogenesis causes oxidation of various important structural and functional proteins and forms protein carbonyls, which serve as markers of oxidative stress, leading to the development/onset of several diseases, including ulceative colitis and cystic fibrosis [48]. Liver damage also leads to excess extracellular proteins accumulation, especially collagen and increased hydroxyproline as observed in liver fibrosis [49]. Iron-dextran injection considerably increased lipid peroxidation (74%), protein carbonyl (155.16%) and hydroxyproline (137.99%) content in liver homogenates compared with normal mice. When treated with PNME, the level of thiobarbituric acid reactive substance (TBARS) was substantially reduced (Fig 6A); however, two other liver damage markers were also found to be arrested significantly with increasing doses (Fig 6B and 6C) in a manner superior to that of the standard desirox. Thus, PNME treatment significantly overcomes hepatic injury/fibrosis in iron-intoxicated mice, indicating the hepato-ameliorating potency of the fruit extract.

#### Histopathological Studies

**Fig 7:**
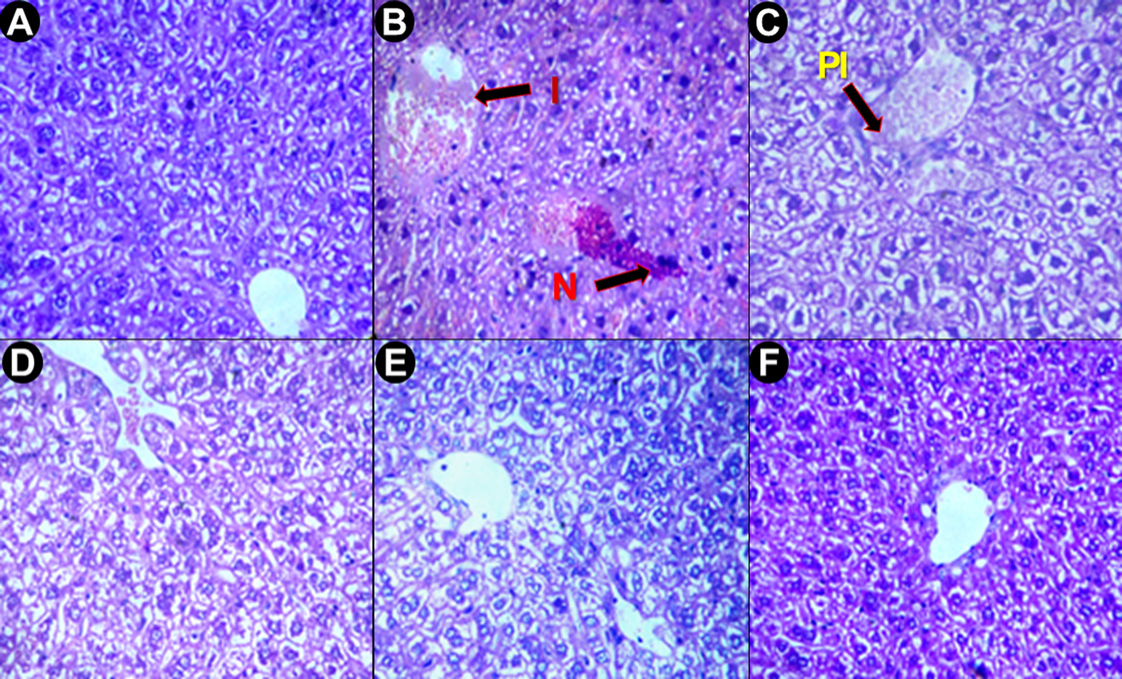
Microscopic observation of mouse liver sections stained with hematoxylin and eosin at 400x. **A**. Liver section from control mice with normal cytoarchitecture. **B**. Iron-overloaded (iron dextran, 100 mg/kg b.w.) liver section shows degeneration of cellular boundaries, fatty ballooning deterioration, inflammation **(I)**, and necrosis **(N)**. **C**. Liver section of S50 shows better cytostructure with minor inflammation **(PI)**. **D**. Liver section of S100. **E**. Liver section of S200. **F**. Desirox-treated liver section shows a reduced necrotic area.

**Fig 8:**
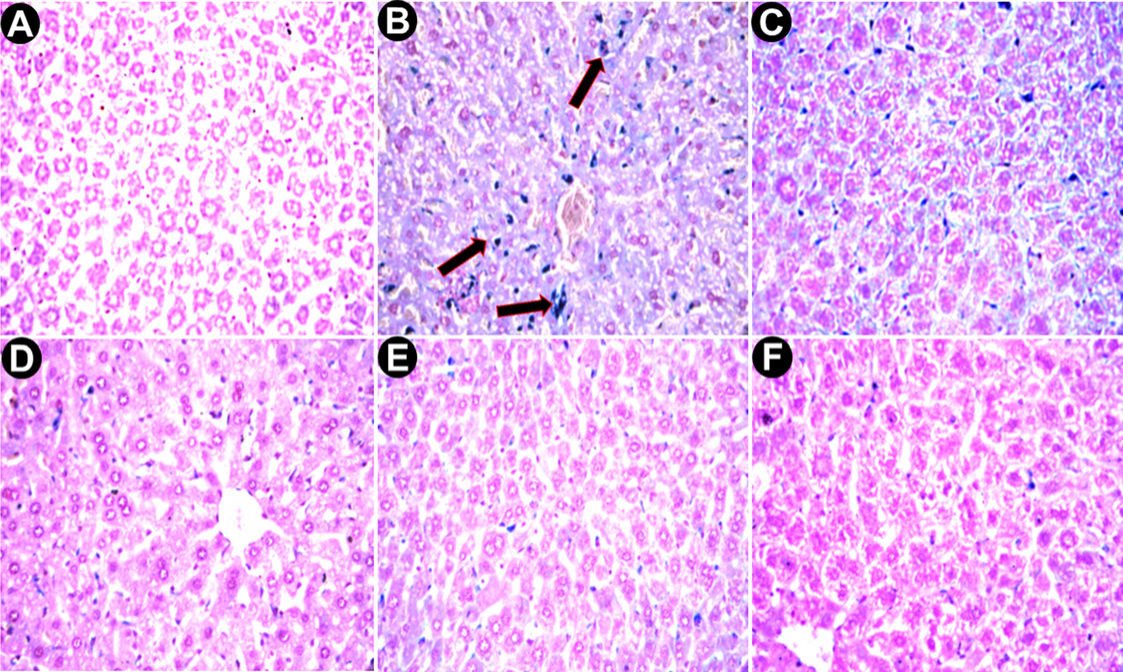
Microscopic observation of liver sections stained with Perls’ Prussian blue at 400x. **A**. Liver section from control mice with normal hemosiderin deposition patch (very low). **B**. Liver section from the C group showing excess amount of blue patches (indicated with arrows). **C**. Liver section from the S50 group. **D**. Liver section from the S100 group. **E**. Liver section from the S200 group. **F**. Desirox-treated liver section shows gradual reduction of blue patches designate significant removal of iron.

**Fig 9:**
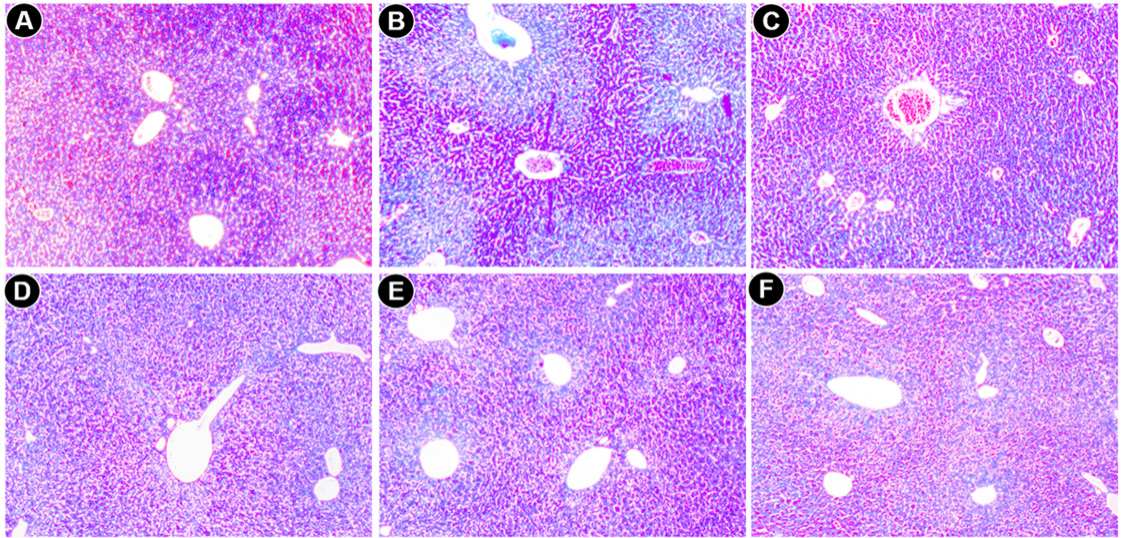
Microscopic observation of liver sections stained with Masson’s trichrome at 100x. A. Liver section from control mice showing no indication of fibrosis. **B**. Liver section of group C exhibiting significant accumulation of collagen (Blue) with elongated fibrous septa. **C**. Liver section from the S50 group shows negligible fibrosis. **D**. Liver section from the S100 group and S200 group (E) depicted similar liver architecture improvement as Desirox-treated group (F).

Histological experiments, a gold standard for the assessment of the degree of hepatic injury, were performed alongside other biochemical tests. The liver sections of normal mice displayed a normal cellular architecture with a prominent nucleus inside cytoplasm and a prominent central vein without cellular infiltration (Fig 7A), whereas saline treated iron-dextran group exhibited abrupt cytological changes, including inflammation, ballooning degeneration, loss of cellular boundaries and hepatocellular necrosis (Fig 7B). In contrast, the liver sections from PNME-treated mice groups displayed evidence of reduced pathogenesis, attenuation of the pathological changes and gradual reversal to normal cyto-architecture, with a higher dosage presenting restoration against iron overload-induced hepatic damage (Fig 7C–7E). An improved liver section morphology was observed in S200 (Fig 7F), which was quite similar to the improvement observed in the desirox-treated group. Another detrimental effect of excess iron in liver is deposition of iron in the form crystalline ferritine and amorphous hemosiderin. Iron released from denatured ferritin, ferric oxide (unused iron) as well as broken hemoglobin formed a complex to store the iron known as hemosiderin. The iron within the deposits of hemosiderin is poorly available to the body and tissue sections stained with Perls’ Prussian blue is commonly used to detect its deposition in liver tissue as blue patches. Light microscopic observation of liver sections of saline treated mice (group B) exhibited elevated deposition of hemosiderin (Fig 8B) compared with normal mice (Fig 8A). These changes also activated stellate cells in periportal zones, which enhanced the production of collagen [50]. In contrast treatment with PNME exhibited a gradual reduction of blue patches (hemosiderin deposition) (Fig 8C, 8D and 8E). PNME exhibited an effect almost parallel to that of the desirox-treated group at S200 (Fig 8F). Additionally, chronic damage in liver due to excessive iron deposition leads to the liver fibrosis characterized by the collapse of the hepatic parenchyma and its substitution with a collagen-rich tissue. A liver biopsy is considered the gold-standard method for the assessment of liver fibrosis [51]. The trichrome stain is performed to assess the degree of fibrosis in liver by staining the nuclei black; cytoplasm red and collagen blue. The microscopic observation suggested that the liver section of control mice revealed normal lobular architecture and a normal distribution of collagen (Fig 9A). From the liver section of iron-overloaded mice it is evident that the normal architecture of the liver is destroyed and the nodules surrounded by accumulated collagen indicating fibrous cirrhotic (Fig 9B). However, after treatment with PNME, a gradual decrease in the degree of collagen deposition was observed (Fig 9C, 9D and 9E). As evident from the other biopsy result, the highest dose of PNME (S200) revealed instances comparable to that of the desirox-treated group (Fig 9F). Overall, histopathological studied indicated that the PNME-treated group produced a dose-dependent normalization of the cyto-architecture, which signifies the in situ evidence of ameliorating effect of the extract in the iron overload-induced liver toxicity.

#### Liver iron content and serum ferritin levels

**Fig 10:**
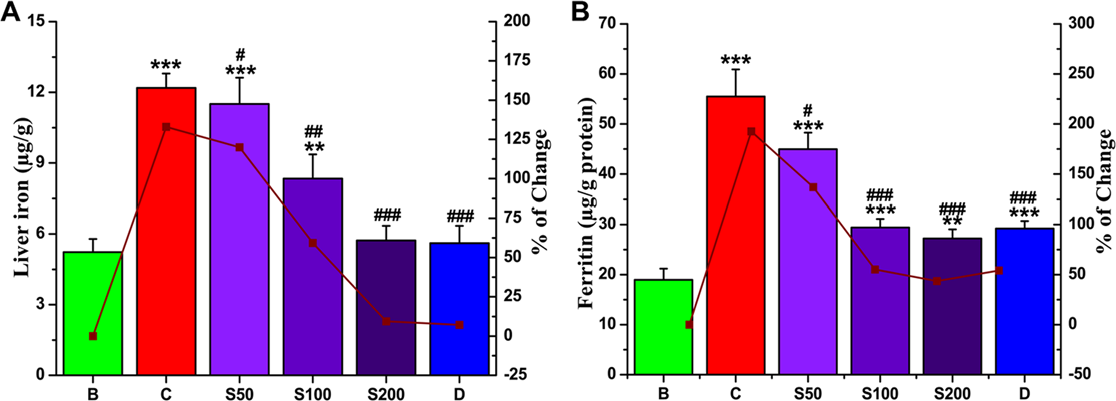
Iron removal potential of PNME. **A.** Iron content in liver, **B.** Ferritin level in serum. Mouse groups (blank, B; control, C; 50 mg/kg b.w. PNME, S50; 100 mg/kg b.w. PNME, S100; 200 mg/kg b.w. PNME, S200; desirox group, D) was treated as described in ‘Experimental design and tissue preparation’ section. The values are expressed as the mean ± S.D. (n = 6). *p < 0.05, **p≤ 0.01, ***p≤ 0.001 compared with blank and ^#^p < 0.05, ^##^p < 0.01, ^###^p≤ 0.001 compared with control.

On the basis of the fact that 5/6 of the surfeited iron in our body is settled in the liver, most procedures seek to measure liver iron levels for diagnosis. Ferritin generally stores excess iron to prevent free iron-mediated oxidative damage to cellular components [52]. The serum ferritin level is one of the main biomarkers for the detection of iron overload-induced hepatic toxicity, as the ferritin content in the blood is indirectly related to the amount of liver iron. The level of liver iron content (132.97%) and serum ferritin content (192.57%) was increased in the iron-intoxicated mice when compared with the normal mice. Upon treatment with PNME, dose-dependent reductions in liver iron content (Fig 10A) as well as serum ferritin concentrations (Fig 10B) were observed. The values were equal to the standard; thus supporting the iron chelating potency of PNME.

#### Iron release from ferritin

**Fig 11:**
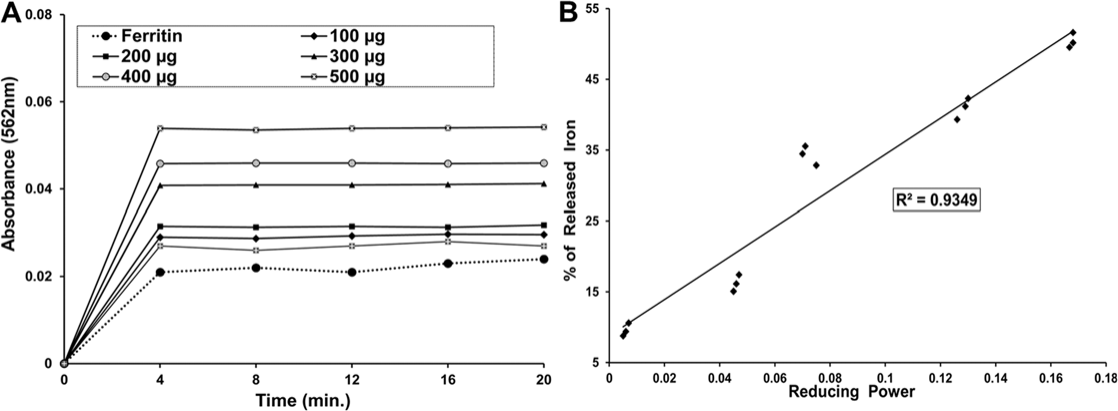
Iron release from ferritin. **A.** Iron release from ferritin **B.** Correlation between reductive released of iron with reducing power of PNME.

Various readily available iron chelator drugs can be administered to normalize the condition generated by iron overload but many of them suffer from limited binding capacity towards ferric iron (Fe^3+^). Therefore, reducing agents, like ascorbic acid, are additionally supplemented to enhance the accessibility of storage iron to chelators [53]. The dose dependent increase in the formation of the ferrous-ferrozine complex [(Fe(ferrozine)_3_)^2+^] was measured to quantify the efficiency of PNME in releasing the reduced iron from ferritin. In the absence of PNME, minor quantities of (Fe(ferrozine)_3_)^2+^ complex formed; however, it was increased significantly with time after the addition of PNME in a dose-dependent manner (Fig 11A). The percentage of iron released from ferritin significantly correlates (R^2^ = 0.9011) with the reducing power of the extract (Fig 11B), thereby confirming the efficacy of PNME as an iron chelating agent.

### Probable phytocompounds identification and HPLC standardization of PNME

In recent years, several edible fruits with natural antioxidants have attracted considerable attention because of their potentially beneficial effects on prevention as well as cure of disease in humans. The medicinal properties of fruits are usually attributed to the active compounds present in them, which can easily be absorbed by our system and are safe for post-consumption symptoms. Therefore, edible fruits have become a major field of interest for the investigation of their diverse pharmacological properties. Qualitative phytochemical analysis of PNME has revealed the presence of phenolics, flavonoids, carbohydrates, terpenoids, tannins, glycosides, anthraquinones, ascorbic acid and alkaloids (Table 3). However, quantitative analysis found that among the phytochemicals, phenolics, flavonoids and alkaloids are present in higher amounts compared with the others (Table 3). Substantially higher amounts of phenolic compounds, subsequent flavonoids and even alkaloids indicate that this fruit represents a source of promising antioxidants, which, as has been well documented, could act as the basis of drug development against various ailments.

**Table 3:** Phytochemical analysis of PNME.

Sample	Tests	Phytochemicals									
		Phenol	Flavonoid	Tannin	Carbohydrate	Ascorbic acid	Alkaloid	Anthra	Terpen	Gly	Sap
PNME	**Qualitative**	+	+	+	+	+	+	+	+	+	-
	**Quantitative**	331.62 ± 4.21	155.06 ± 1.36	19.17 ± 0.03	33.01 ± 0.11	10.03 ± 0.23	114.52 ± 1.95	ND	ND	ND	ND

Anthra- Anthraquinone, Terpen-Terpenoids, Gly-Glycoside, Sap-Saponin. Total phenolics (mg/100 mg extract gallic acid equivalent), Total flavonoids (mg/100 mg extract quercetin equivalent), Tannin (mg/100 mg extract catechin equivalent), Carbohydtrate (mg/100 mg extract glucose equivalent), Ascorbic acid (mg/100 mg extract L-ascorbic acid equivalent), Alkaloid (mg/100 mg extract reserpine equivalent), “+” represents presence of the phytoconstituent; “-” represents absence of the phytoconstituent. “ND” represents Not Determined.

**Fig 12:**
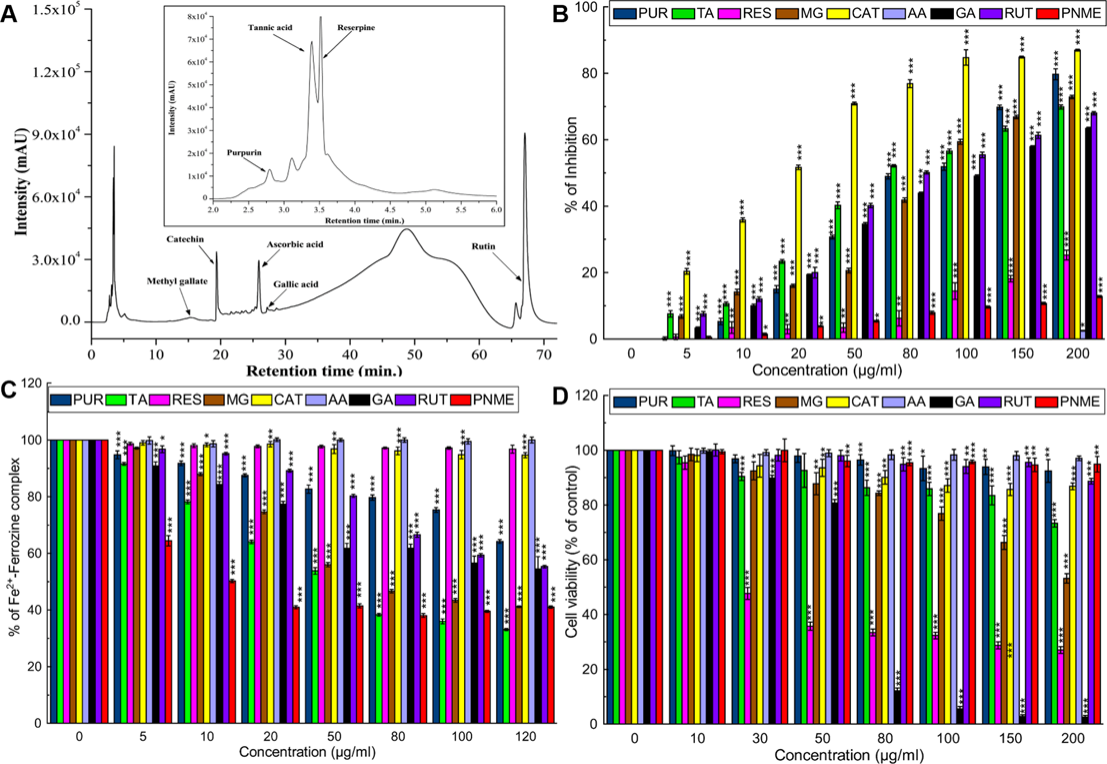
HPLC chromatogram of PNME, free radical scavenging, iron chelation and cytotoxic activity of the identified compounds of PNME. **A.** HPLC chromatogram with expanded view between retention time of 2–6 min (inset). The marked peaks denote the retention of peaks matched with the retention of the respective standard phytochemicals in the same condition. **B**. Hydroxyl radical scavenging assay, **C.** Iron chelation assay **D.** Cytotoxicity assay.

To identify the existing phytochemicals in PNME, HPLC analysis was performed and the retention time of the main peaks was compared with the standard phytocompounds in the identical condition. Major peaks with retention times 2.8, 3.39, 3.52, 15.39, 19.38, 25.98, 27.20, 67.18 min appeared on the HPLC chromatogram of PNME that matched the chromatographic patterns of purpurin, tannic acid, reserpine, methyl gallate, catechin, ascorbic acid, gallic acid and rutin, respectively (Fig 12A). Probable identified phytochemicals were screened for their *in vitro* hydroxyl radical scavenging, iron chelation potentials and cytotoxicity against human normal fibroblast cells (WI-38). Free radical scavenging was assessed using a hydroxyl radical scavenging assay, as an excess iron condition induces hydroxyl radicals that result in lipid peroxidation-mediated hepatotoxicity. Except for ascorbic acid, all compounds exhibited promising hydroxyl radical scavenging activity (Fig 12B). Among the identified compounds, tannic acid was found to be the most potent iron chelator, followed by methyl gallate, gallic acid, rutin and purpurin. Reserpine, catechin and ascorbic acid failed to show any activity (Fig 12C). On the other hand, PNME exhibited excellent iron chelation activity. This result indicates that the unidentified compounds of PNME are also responsible for the improved iron chelation activity of PNME. The cytotoxicity of the identified compounds on the normal fibroblast cells indicated that gallic acid is most toxic towards normal cells followed by reserpine, methyl gallate, tannic acid, catechin, rutin and purpurin. Most importantly, PNME did not exhibit any cytotoxicity against normal cells (Fig 12D). From the reported literature, it was found that purpurin, methyl gallate, gallic acid, rutin, tannic acid, ascorbic acid, catechin are potential antioxidants, metal chelators thereby inhibit lipid peroxidation, which also support their hepato-ameliorating potentials [54–57]. On the other hand, reserpine as well as its derivatives have been reported to have antioxidant potential [58]. Rutin, a citrus flavonoid glycoside, is found in many fruits, such as oranges, grapefruits, lemons, cranberries and even other species of *Prunus*. Apart from its antioxidant, metal chelation and protective effects on hepatotoxicity, it also exhibits anti-inflammatory activity [59]. From the cytotoxicity point of view, some of the individual compounds are toxic to normal cells; however, when in combination, their toxic effect is nullified, possibly due to the presence of other nontoxic compounds in the extract. This possibility is supported by the fact that the extract (PNME) is a mixture of several compounds and their synergistic effect is responsible for antioxidant activity and its ameliorating activity against iron-induced hepatotoxicity.

## Conclusions

The results demonstrated high efficacies of the 70% methanolic extract of *P*. *nepalensis* in scavenging free radicals, inhibiting reactive free radicals and chelating iron, which established its therapeutic use as a functional food that may effectively treat several diseases, with liver disease being the most important. Phytochemical screening also indicated the presence of several bioactive phytocompounds and natural antioxidants that are responsible for its overall activity. Cytotoxicity studies suggested that a single molecule may have greater activity but also may possess toxicity towards normal cells. Therefore, combination therapy has a greater impact without affecting normal cells. As, several other causes of liver toxicity such as drug induced, alcohol overloaded, viral hepatitis, nonalcoholic fatty liver follow similar pathway [60,61] for liver toxicity and PNME can be used to ameliorate them. But, presently it is hard to comment whether PNME would work similar manner on other types of hepatotoxicity or not. So, further experiments in other animal models are required to fully evaluate the antioxidant and hepato-ameliorating potentials along with the complete characterization of all bioactive compounds present in this wild edible fruit. This information will establish its use in combinatorial therapy and innovative drug delivery systems, thus making it a favorable candidate to manage iron overload-mediated oxidative stress and hepatotoxicity.

## Supporting Information

S1 ARRIVE Checklist The ARRIVE Checklist. (DOCX)
