# Supplementary material for: Wild Edible Fruit of Prunus nepalensis Ser. (Steud), a Potential Source of Antioxidants, Ameliorates Iron Overload-Induced Hepatotoxicity and Liver Fibrosis in Mice
Source: PLoS One. 2015 Dec 3;10(12):e0144280. doi: 10.1371/journal.pone.0144280 (PMC4669143; doi:10.1371/journal.pone.0144280)
Supplement: S1 ARRIVE Checklist — (DOCX) [file pone.0144280.s001.docx]

**The ARRIVE Checklist**

**TITLE**

**1 Provide as accurate and concise a description of the content of the article as possible.**

Wild edible fruits of *Prunus nepalensis* Ser. (Steud) a potential source of antioxidants, ameliorate iron-overload induced hepatotoxicity and liver fibrosis in mice

**ABSTRACT**

**2 Provide an accurate summary of the background, research objectives (including details of the species or strain of animal used), key methods, principal findings, and conclusions of the study.**

The antioxidant and restoration potentials of hepatic injury by *Prunus nepalensis* Ser. (Steud), a wild fruit plant from the North-Eastern region of India was investigated. The fruit extract (PNME) exhibited excellent antioxidant and reducing properties and also scavenged 2,2-diphenyl-1-picrylhydrazyl (DPPH) radical (IC_50_ = 30.92 ± 0.40 μg/ml). Additionally, the free radical scavenging activities of the extract was assessed by the scavenging of different reactive oxygen and nitrogen species and demonstrated promising scavenging potency. Moreover, the extract revealed an exceptional iron chelation capacity with an IC_50_ of 25.64 ± 0.60 μg/ml. The extract showed signiﬁcant improvement of hepatic injury against iron (100 mg/kg) induced liver damage in mice in a dose dependent manner which also supported by different histopathological studies. The phytochemical composition and their identification by high performance liquid chromatography (HPLC) confirmed the presence of purpurin, tannic acid, reserpine, methyl gallate, catechin, ascorbic acid, gallic acid and rutin. Identified compounds were checked for their individual radical scavenging and iron chelation activity; some of them exhibited excellent radical scavenging as well as iron chelation properties, but most of them were toxic towords the normal cells (WI-38). On the other hand crude PNME was found to be totally nontoxic to normal cells, suggesting its feasibility as a source of safe oral drugs. The above study suggests that different phytochemicals in PNME contributed to its free radical scavenging as well as iron chelation activity but further studies are required to determine the path way in which PNME act to treat iron overload diseases.

**INTRODUCTION**

**Background**

**3 a. Include sufficient scientific background (including relevant references to previous work) to understand the motivation and context for the study, and explain the experimental approach and rationale.**

**b. Explain how and why the animal species and model being used can address the scientific objectives and, where appropriate, the study’s relevance to human biology.**

Tissues, irrespective of human or murine origin, when subjected to oxidative stress of any kind face detriments by virtue of tainted/manipulated biomolecules further leading to critical failure of biological functions. Similarly, excess iron dumping in hepatic tissues due to elevated plasma iron (because of hemolysis, dietary iron overload, inflammatory syndrome, blood transfusion, diabetic conditions or chronic liver diseases) instigate iron-mediated-free radical-induced oxidative and/or nitrosative stresses. For analyzing the preventive or curative approaches and to gain an understanding of the mechanism of oxidative pathologic processes in liver diseases caused due to iron-overload, the availability of human samples is extremely restricted and on some grounds, unethical. In an order to mimic the same conditions, we have considered the widely practiced and accepted, murine model where the experiments are conducted under strict ethical guidelines and scientific sense. Moreover, the ease in handling the murine model and the tissues obtained from it, further facilitates our way of understanding the answer to the raised scientific question. We believe that obtaining the experimental results from iron-overloaded mice liver will give us an adequate milieu to understand the mechanistic role in liver hemochromatosis and hemosiderosis along with oxidative stress and lipid peroxidation in humans.

**Objectives**

**4 Clearly describe the primary and any secondary objectives of the study, or specific hypotheses being tested.**

**Primary objectives:**

- To develop an orally administrable safer and better drug from a wild edible fruits of *Prunus nepalensis*, for amelioration of iron-induced liver toxicity

**Secondary objectives:**

- To understand the effect of PNME in protecting mice liver from hepatic damage due to iron-overload.

**METHODS**

**Ethical statement**

**5 Indicate the nature of the ethical review permissions, relevant licenses (e.g. Animal [Scientific Procedures] Act 1986), and national or institutional guidelines for the care and use of animals, that cover the research.**

All experiments related to the animals (Swiss albino mice) were performed after obtaining approval from the Institutional Animal Ethics Committee, with certified regulations of the Committee for the Purpose of Control and Supervision of Experiments on Animals (CPCSEA), Ministry of Environment and Forest, Govt. of India (Bose Institute Registration. No. 95/1999/CPCSEA).

**Study design**

**6 For each experiment, give brief details of the study design, including:**

**a. The number of experimental and control groups.**

**b. Any steps taken to minimise the effects of subjective bias when allocating animals to treatment (e.g., randomisation procedure) and when assessing results (e.g., if done, describe who was blinded and when).**

**c. The experimental unit (e.g. a single animal, group, or cage of animals).**

**A time-line diagram or flow chart can be useful to illustrate how complex study designs were carried out.**

a. A total of 36 mice were divided into six groups comprising six mice in each group. One group was labeled as a blank (B) and received normal saline only. The other five groups were injected intraperitoneally every alternate day with a 100 mg/kg b.w. dose of iron-dextran for 10 days, i.e. 5 doses in 10 days. One iron-dextran group (C) was orally administered with normal saline and other four groups were orally treated with 50 mg/kg b.w. (S50), 100 mg/kg b.w. (S100), 200 mg/kg b.w. (S200) PNME and 20 mg/kg b.w. desirox (D), respectively, for 21 days starting the day following the first iron-dextran injection.

b. All mice selected for the study were randomly selected from a male population.

c. A group of 6 mice per cage were considered for acquiring data for a single dose (n=6).


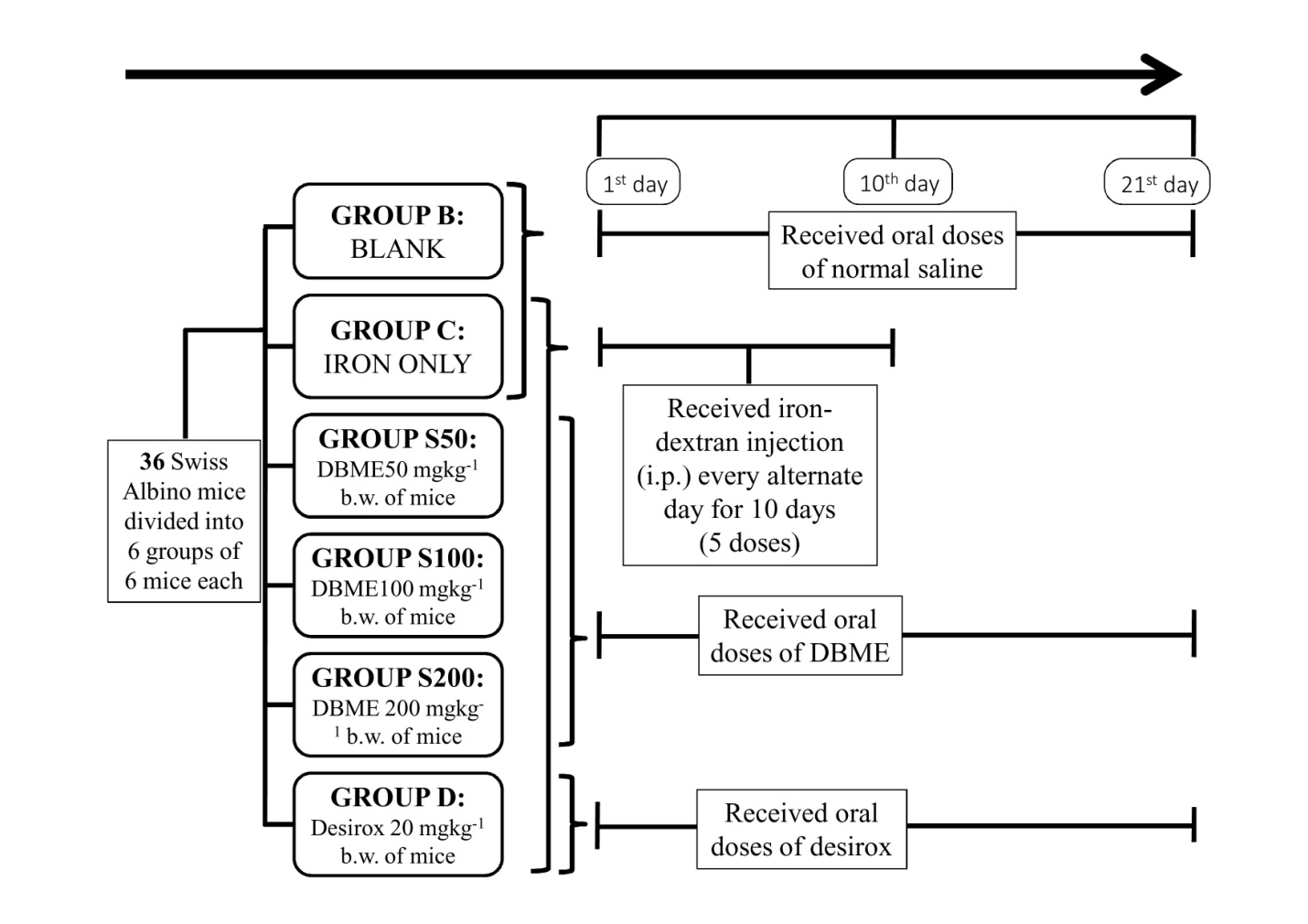


**Experimental procedures**

**7 For each experiment and each experimental group, including controls, provide precise details of all procedures carried out. For example:**

**a. How (e.g., drug formulation and dose, site and route of administration, anaesthesia and analgesia used [including monitoring], surgical procedure, method of euthanasia). Provide details of any specialist equipment used, including supplier(s).**

**b. When (e.g., time of day).**

**c. Where (e.g., home cage, laboratory, water maze).**

**d. Why (e.g., rationale for choice of specific anaesthetic, route of administration, drug dose used).**

a. Route of administration:

Iron-dextran was injected intra-peritoneally using 1 ml disposable syringes of needle size 0.45×13 mm/26G×1/2, by usual procedure and adequate expertise. Normal saline, PNME and Desirox were administered orally using appropriate feeding needles.

b., c., d. All mice were sacrificed between 5 pm to 6 pm under inhaled anesthesia ethyl ether for 10 minutes as per approved animal protocol in the laboratory. Blood was collected by cardiac puncture followed by immediate cervical dislocation of each animal. Entire liver was dissected out, washed with ice-cold saline and divided into different tissue sections as required.

**Experimental animals**

**8 a. Provide details of the animals used, including species, strain, sex, developmental stage (e.g., mean or median age plus age range), and weight (e.g., mean or median weight plus weight range).**

**b. Provide further relevant information such as the source of animals, international strain nomenclature, genetic modification status (e.g. knock-out or transgenic), genotype, health/immune status, drug- or test naıve, previous procedures, etc.**

a. Species- *Mus musculus*, strain- Swiss albino, sex- male, age range- post weaning 3 to 4 weeks, mean weight- 20±2 g

b. Source of animals- Chittaranjan National Cancer Institute (CNCI), Kolkata India. International strain nomenclature-N/A, Genetic modification status- None, genotype- out bred animal, Health/immune status- Good, Drug- or test naïve- None, previous procedures- None

**Housing and husbandry**

**9 Provide details of:**

**a. Housing (e.g., type of facility, e.g., specific pathogen free (SPF); type of cage or housing; bedding material; number of cage companions; tank shape and material etc. for fish).**

**b. Husbandry conditions (e.g., breeding programme, light/dark cycle, temperature, quality of water etc. for fish, type of food, access to food and water, environmental enrichment).**

**c. Welfare-related assessments and interventions that were carried out before, during, or after the experiment.**

a. Housing- community animal house under aseptic conditions; type of cage-ventilated; bedding- sterile rice husk bedding (height-1 inch). Number of cage companions-6 same experimental group mice.

b. Husbandry conditions- maintained under a constant 12 h dark/light cycle (9 pm-9 am) at an environmental temperature of 22±2°C; food and water- normal laboratory pellet diet and water *ad libitum*.

c. All animal house conditions including the health of experimental mice, feeding conditions, temperature, etc. were checked three times a day by concerned person.

**Sample size**

**10 a. Specify the total number of animals used in each experiment and the number of animals in each experimental group.**

**b. Explain how the number of animals was decided. Provide details of any sample size calculation used.**

**c. Indicate the number of independent replications of each experiment, if relevant.**

a. A total number of 36 mice were divided into six experimental groups comprising six mice in each group.

b. As the entire set up required experiments to be conducted 6 times, in addition to 6 experimental conditions (including control and treatment cages-as mentioned in #6a), 6×6 = 36 mice were randomly chosen.

c. Not applicable.

**Allocating animals to experimental groups**

**11 a. Give full details of how animals were allocated to experimental groups, including randomisation or matching if done.**

**b. Describe the order in which the animals in the different experimental groups were treated and assessed.**

a. 36 mice of similar weight (20±2 g) and age group (3-4 weeks old) were randomly selected by the laboratory assistant (who was not directly associated with the experiment).

b. The order of treatment is as follows:

Normal saline → PNME (from lower to higher doses) → Desirox.

**Experimental outcomes**

**12 Clearly define the primary and secondary experimental outcomes assessed (e.g., cell death, molecular markers, behavioural changes).**

**Primary experimental outcomes assessed:**

The experimental animals were assessed whether they will recover from iron induced oxidative stress after the treatment of test drug (PNME) as compared to the group not receiving the test drug.

**Secondary experimental outcomes assessed**

The experimental animals were assessed for the changes in their liver iron content, liver enzyme parameters, hepatotoxicity parameters after the treatment of test drug (PNME) as compared to the group not receiving the test drug.

**Statistical methods**

**13 a. Provide details of the statistical methods used for each analysis.**

**b. Specify the unit of analysis for each dataset (e.g. single animal, group of animals, single neuron).**

**c. Describe any methods used to assess whether the data met the assumptions of the statistical approach.**

All data are reported as the mean ± SD of six measurements. Statistical analysis was performed using KyPlot version 2.0 beta 15 (32 bit) and Origin professional 6.0. Comparisons among groups were made according to pair *t*-test. In all analyses, a *p* value of <0.05 was considered significant.

**RESULTS**

**Baseline data**

**14 For each experimental group, report relevant characteristics and health status of animals (e.g., weight, microbiological status, and drug- or test-naıve) before treatment or testing (this information can often be tabulated).**

All animals analyzed were in good health.

**Numbers analysed**

**15 a. Report the number of animals in each group included in each analysis. Report absolute numbers (e.g. 10/20, not 50%).**

**b. If any animals or data were not included in the analysis, explain why.**

Please refer to point #6 for the same.

**Outcomes and estimation**

**16 Report the results for each analysis carried out, with a measure of precision (e.g., standard error or confidence interval).**

All data are reported as the mean ± Standard Deviation of six measurements. The related results with appropriate statistics are as follows:

**Table** The effect of PNME on serum markers (ALAT, ASAT, ALP, bilirubin) in iron overloaded mice

| **Treatment** | **ALAT (Unit/l)** | **% Change** | **ASAT (Unit/l)** | **% Change** | **ALP (Unit/l)** | **% Change** | **Bilirubin (mg/dl)** | **% Change** |
| --- | --- | --- | --- | --- | --- | --- | --- | --- |
| **B** | 15.25±1.58 | - | 67.61± 5.78 |  | 33.08±3.71 |  | 1.51±0.09 |  |
| **C** | 45.23±2.45^X3^ | 196.63 | 167.62±4.64 ^X3^ | 147.92 | 132.51±6.42 ^X3^ | 300.58 | 3.28±0.20 ^X3^ | 116.29 |
| **S50** | 39.52±1.21 ^X3Y2^ | 159.16 | 129.01±9.30 ^X3^ | 90.82 | 123.72±3.59^X3Y1^ | 274.01 | 3.06±0.19 ^X3^ | 101.36 |
| **S100** | 28.82±2.26 ^X3Y3^ | 88.99 | 99.81±3.10 ^X3 Y3^ | 47.63 | 99.43±6.27 ^X3Y3^ | 200.58 | 2.85±0.30^X3Y2^ | 87.78 |
| **S200** | 19.21±2.18 ^X1Y3^ | 25.95 | 75.18±4.81 ^Y3^ | 11.19 | 66.35±6.47 ^X3Y3^ | 100.58 | 2.61±0.22^X3Y2^ | 71.95 |
| **D** | 23.55±1.09 ^X3Y3^ | 52.80 | 79.35±4.37 ^X1 Y3^ | 17.36 | 61.01± 2.44 ^X3Y3^ | 84.44 | 1.68 0.15^Y3^ | 10.97 |

Values are mean ± SD of six observations.

X1: *p<*0.05, X2:*p<*0.01 and X3: *p<*0.001 significant difference from normal mice (B) group

Y1: *p<*0.05,Y2:*p<*0.01 and Y3: *p*<0.001significant difference from iron overloaded (C) group

**
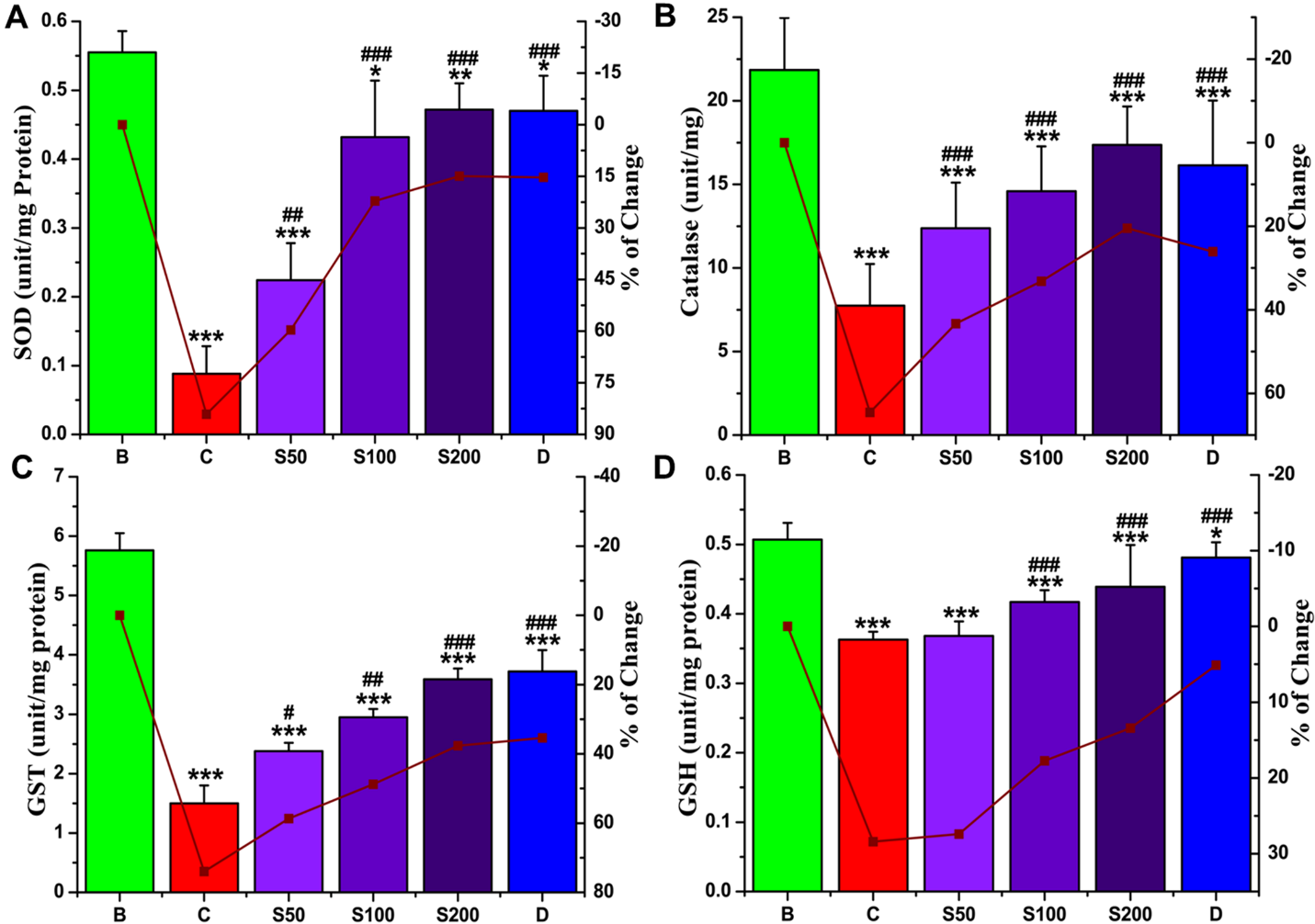
**

**Figure Effect of PNME on the antioxidant enzymes against iron overload induced hepatic injury in mice.** A. SOD, B. Catalase, C. GST, D. GSH. Mice were randomly divided into six groups (blank, B; control, C; 50 mg/kg b.w. PNME, S50; 100 mg/kg b.w. PNME, S100; 200 mg/kg b.w. PNME, S200; desirox group, D) and treated as described in ‘2.5.1’ section. Values are expressed as mean ± SD of six mice. *p < 0.05, **p≤ 0.01, ***p≤ 0.001 compared with blank and #p < 0.05, ##p≤ 0.01, ###p≤ 0.001 compared with control.


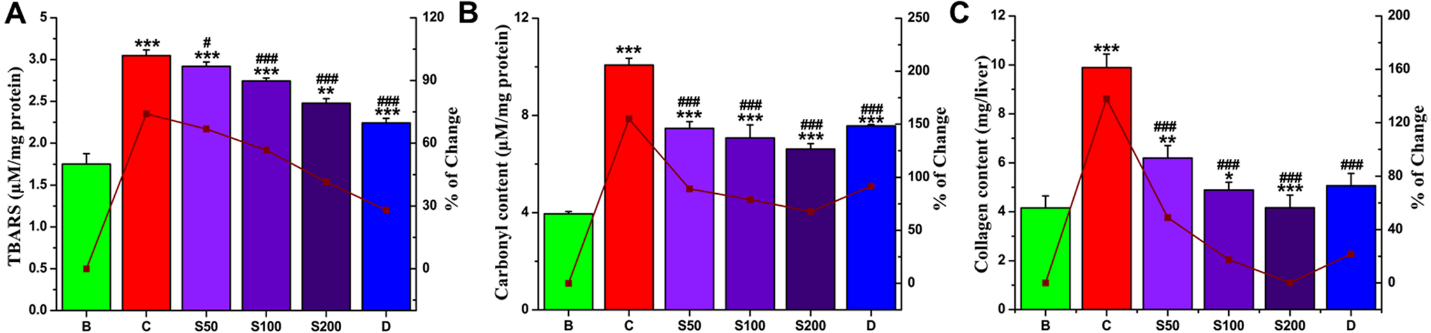


**Figure Effect of PNME on biochemical parameters.** (A) Hepatic lipid peroxidation levels, (B) protein oxidation levels, (C) collagen content. Mice were randomly divided into six groups (blank, B; control, C; 50 mg/kg b.w. PNME, S50; 100 mg/kg b.w. PNME, S100; 200 mg/kg b.w. PNME, S200; desirox group, D) and treated as described in ‘experimental design’ section. Values are expressed as mean ± SD of six mice. *p < 0.05, **p < 0.01, ***p < 0.001 compared with blank and #p < 0.05, ###p < 0.001 compared with control.


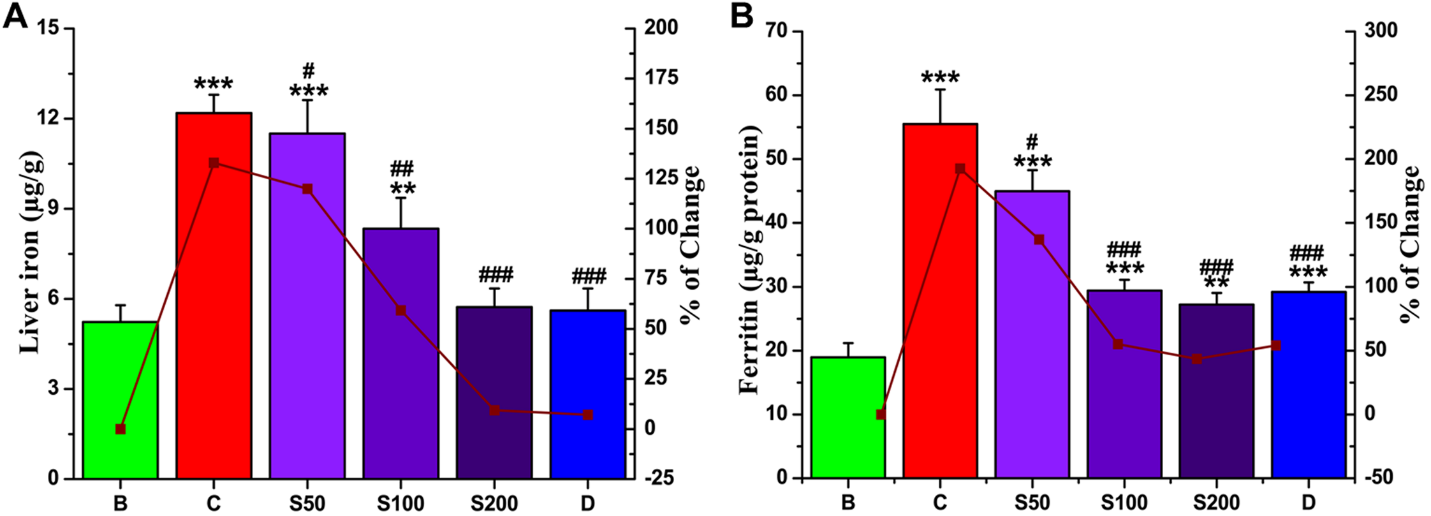


**Figure** **Effect of PNME on hepatic iron content and serum ferritin level.** (A) hepatic iron content, (B) serum ferritin level. Mice were randomly divided into six groups (blank, B; control, C; 50 mg/kg b.w. PNME, S50; 100 mg/kg b.w. PNME, S100; 200 mg/kg b.w. PNME, S200; desirox group, D) and treated as described in ‘experimental design’ section. Values are expressed as mean ± SD of six mice. **p < 0.01, ***p < 0.001 compared with blank and #p < 0.05,##p < 0.01, ###p < 0.001 compared with control.

**Adverse events**

**17 a. Give details of all important adverse events in each experimental group.**

**b. Describe any modifications to the experimental protocols made to reduce adverse events.**

No adverse events were witnessed.

**DISCUSSION**

**Interpretation/scientific implications**

**18 a. Interpret the results, taking into account the study objectives and hypotheses, current theory, and other relevant studies in the literature.**

**b. Comment on the study limitations including any potential sources of bias, any limitations of the animal model, and the imprecision associated with the results.**

**c. Describe any implications of your experimental methods or findings for the replacement, refinement, or reduction (the 3Rs) of the use of animals in research.**

a. The alleviation of free iron induced oxidative stress and hepatotoxicity in mice by PNME, which is also far better as compared to the standard drug desirox, confirms its *in vivo* free radical scavenging as well as hepatoprotective activity. This activity of PNME may result from the potential to upregulated antioxidant enzymes and chelate free redox active iron followed by its excretion from the body.

b. There might be strain-specific differences in the drug response, which were not assessed in this study.

c. Due to the lack of cell lines which can be used for iron overloading and which resembles the hemochromatosis as well as hemosiderosis, an *in vitro* study design is not currently feasible.

**Generalisability/translation**

**19 Comment on whether, and how, the findings of this study are likely to translate to other species or systems, including any relevance to human biology.**

This study is designed to obtain a background of knowledge of any protection induced by PNME against hepatic damage caused due to iron overload. It is obvious that the hepatic constitution in humans and mice are identical, but since in the present experimental set up it is impossible to acquire adequate number and quantity of human tissue samples to conduct a similar analysis due to medical and ethical constraints, delivering a comprehensive statement about the efficacy of PNME cannot be stated. Moreover due to the possible similarities between the two species, mouse is considered as the nearest accessible experimental model.

**Funding**

**20 List all funding sources (including grant number) and the role of the funder(s) in the study.**
